# Epidemiology of osteoporosis and its determinants in physically active Majorcan elderly

**DOI:** 10.31138/mjr.31.1.42

**Published:** 2020-03-31

**Authors:** Antonio Juan, Guillem Frontera, Ana Paula Cacheda, Inmaculada Ros, Javier Narváez, Bartolomé Marí, Joan Miquel Nolla

**Affiliations:** 1 Servicio de Reumatología, Hospital Universitari Son Llàtzer, Palma de Mallorca, Spain,; 2 Unidad de Investigación, Hospital Universitari de Son Espases, Palma de Mallorca, Spain; 3 Servicio de Reumatología, Hospital Universitari de Bellvitge, L’Hospitalet de Llobregat, Spain,; 4 IDIBELL, L’Hospitalet de Llobregat, Spain,; 5 Unidad de Medicina del Deporte. Institut de Serveis Socials i Esportius. Consell de Mallorca, Palma de Mallorca, Spain

**Keywords:** osteoporosis, prevalence, aged people, bone mineral density

## Abstract

**Objective:**

To estimate the prevalence and distribution of determinants of osteoporosis (OP) in a population of physically active Majorcans over 60.

**Methods:**

Health survey in which consecutive women and men above 60 years old visiting sports facilities during a two-month period were recruited. All underwent a densitometry of the lumbar spine (LS) and femoral neck (FN). Osteoporosis was defined according to the World Health Organization densitometric criteria (T-score <2.5 SD in the LS or FN, and osteopenia if the result was between −2.5 and −1 SD). As osteoporosis shows substantial differences between genders, the study of its determinants was conducted independently for men and women.

**Results:**

The sample included 731 subjects (86% female), with an average age of 70 (SD 5) among men and 65 (8) among women. The overall prevalence of osteoporosis was 35.7% in the LS, 8.9% in the FN and 39.4% in the LS and/or FN. The analysis by gender showed a higher prevalence of osteoporosis in women than in men (43.8 % vs. 11.1%). The presence of osteoporosis increased with age in men and women (7.8% for 61–75 years old vs 22.7% > 75 years old for men and 48.5% for 61–75 years old vs 62.7% > 75 for women).

**Conclusions:**

Densitometric osteoporosis is frequent among physically active elderly population, and higher than expected in a largely sunlight-exposed area.

## INTRODUCTION

Osteoporosis is the most frequent metabolic bone disease. However, since it is asymptomatic, it may not be diagnosed until a clinical event such as a fracture has occurred.^1,2^ It is osteoporotic fractures, not osteoporosis itself, that lead to notable clinical and economic impact.^1,3^

The World Health Organization (WHO) estimates a lifetime risk of osteoporotic fracture at hip, vertebra or wrist of 30 to 40% in developed countries.^1^ The prevalence of osteoporosis in the 27 countries of the European Union among people older than 50 years is 15% (22% in women and 6.6% in men),^3,4^ and the European Prospective Osteoporosis Study (EPOS) estimated a prevalence of osteoporosis of 15% in women 50 to 60 years old and of 45% in women over 70.^5^ In men, the prevalence estimated in EPOS was 2.4% between 50 and 60 years and 17% over 70.^5^

Along with age, gender and body mass index (BMI), bone mineral density (BMD) is a strong risk factor for the occurrence of new bone fractures. Densitometric osteoporosis is an important feature when evaluating the risk of a new fracture. Because BMD in the young healthy population is normally distributed and bone loss occurs with age, osteoporosis increases also with age and thus its prevalence depends on the demographics of the populations studied. Considering a progressive ageing population, it is easy to predict a continuous increment in the consequences of osteoporotic fractures, if the rate of falls is not reduced, eventually placing osteoporotic fractures due to falls among the major health problems.^6^ In the Mediterranean area, the prevalence of low BMD (or densitometric osteoporosis) and associated factors has not been extensively studied. Furthermore, in recent years, life expectancy has been increasing, especially in the Mediterranean area. This has been translated into more elderly people, many of whom are well fitted physically. Being physically active is documented to be one of the healthy behaviours for preventing and managing diseases among older adults.^7^ Thus, it becomes necessary to investigate the particular characteristics of certain diseases (eg, osteoporosis) in this new elderly population profile.

Mallorca is the largest island in the Balearic Islands (with a total population on the islands of 1,128,139 people as of January 2018).^8^ This island is part of Spain, and thus covered by its universal health system, and is located in the Mediterranean, with a mild climate and with the majority of its population following a Mediterranean diet, of known beneficial effects on health, including bone health.^9^

In this work we intend to evaluate the prevalence of densitometric osteoporosis and its determinants in a Majorcan elderly population of physically active men and women. Our aim is to study in detail the prevalence of osteoporosis in a new Mediterranean elderly profile. Our hypothesis was that although this is an essentially healthy and fit population group, the prevalence of low bone mass (OP or osteopenia) would be relevant in both genders, both in the lumbar spine and in the proximal third of the femur.

## METHODS

This was a cross-sectional study conducted for 2 years in Mallorca. The project was part of the Program “*Gent gran en marxa*”, promoted by the Consell Insular de Mallorca, aimed at improving the health and quality of life of a group of elderly people, through the development of a series of activities (dance, gymnastics, among others), and the assessment of the health status and their physical possibilities (cardiovascular study, muscle power, diagnosis of osteoporosis, among other studies). All the people who attended the participating senior centres during the study period were invited to participate in the study and those who accepted were referred to a centre to carry out the interviews and densitometry.

People were referred to the densitometry unit from the *Institut Municipal d’Esports* in Palma de Mallorca and surroundings, and underwent a densitometry of the lumbar spine and femoral neck, in a period of less than 15 days.

Osteoporosis was defined according to the WHO densitometric criteria, ie, osteoporosis was present when the BMD result showed a T-score <2.5 SD in the lumbar spine or femoral neck. Osteopenia was defined as a T-score between -2.5 and −1 SD. A DXA Norland® unit, model XR-46, was used to perform the lumbar spine and femoral neck BMD, previously calibrated and with proper maintenance and calibrations by the supplier. The densitometry was carried out by a qualified technician who had previously been trained in the unit of densitometry, patient positioning and computer support. NHANES III reference values were used. The reference population for both men and women is drawn from the Third National Health and Nutrition Examination Survey (NHANES III).^10^

Participants visited the rheumatologist to complete a clinical history and were asked to fill out a questionnaire to specify the following information: age, gender, morphotype, size and weight, history of maternal hip fracture, history of Colles’ fracture, hip fracture or any previous osteoporotic fracture, hyperthyroidism, diabetes mellitus, Parkinson’s disease, history of cerebrovascular disease.

Other variables collected were: smoking (non-active vs active) and alcohol drinking (not at all vs any) habits, sun exposure (avoidance vs any exposure), physical activity (no planned exercise/activity vs any planned physical activity), number of hours standing, history of corticosteroid or benzodiazepines intake. In women, gynaecological variables were also collected, such as history of breast cancer, pregnancy, breastfeeding, early menopause (before age 42), previous oophorectomy, oral contraceptive intake, and years of menstruation.

### Statistical analysis

For the description of the sample, summary statistics were used. Prevalence was estimated with 95% confidence intervals, assuming a Poisson distribution. Estimates were obtained for the overall sample and by gender and age groups. The sample of men and women were compared regarding basic descriptors. Given the multiple comparisons done, the significance level was adjusted to 0.01/22 = 0.0001.

The potential determinants of osteoporosis were analysed by constructing multivariable logistic regression models, using the diagnosis of osteoporosis by any location as the dependent variable. Models were calculated separately by gender. Demographic characteristics, personal history and known risk factors for osteoporosis were introduced as independent variables. The following aspects were taken into account when including variables in the models: the results of the bivariate analysis (variables with a p<0.250 value), clinical reasoning, and sample size. In the first (saturated) model, we included the variables that obtained a p<0.250 value in the bivariate analysis and tried to explain the diagnosis of osteoporosis with the most parsimonious model or with the fewest independent variables. Models were compared using the information measures of Aikaike and Bayesian (AIC and BIC, respectively).

## RESULTS

The sample consisted of 731 people (632 women and 99 men) with an average age of 70.2 (SD=5.4) years among men and 64.5 (8.9) in females. The socio-demographic characteristics of the sample are presented in *Table 1*.

**Table 1.** Description and distribution of osteoporosis determinants in the sample of physically active elderly Majorcan.

**Characteristic**	**Total (N=731)**	**Men (N=99)**	**Women (N=632)**	p-value^*^

Age in years, mean (SD)	65.3 (8.8)	70.2 (5.4)	64.5 (8.9)	<0.0001

Weight in Kg, mean (SD)	65.0 (10.4)	76.3 (11.2)	63.2 (9.1)	<0.0001

Height in m, mean (SD)	1.58 (0.1)	1.68 (0.1)	1.56 (0.06)	<0.0001

BMI, mean (SD)	26.1 (3.5)	27.2 (3.6)	25.9 (3.5)	0.001

Standing upright >4 h	492 (67.3)	66 (66.7)	426 (67.4)	0.884

Physical activity				
No	68 (9.3)	10 (10.1)	58 (9.18)	0.844
Mild, moderate	386 (52.8)	54 (54.5)	332 (52.5)	
Intense	277 (37.9)	35 (35.3)	242 (38.3)	

Smokers	69 (9.4)	16 (16.2)	53 (8.4)	0.014

Alcoholic habit	87 (11.9)	28 (28.3)	59 (9.3)	<0.0001

Coffee intake	417 (57.0)	64 (64.6)	353 (55.8)	0.100

Sun exposure	526 (72.0)	84 (84.8)	442 (69.9)	0.002

Morphotype				
Asthenic	69 (9.5)	6 (6.1)	63 (10.0)	0.024
Athletic	331 (45.4)	57 (57.6)	274 (43.5)	
Pyknic	329 (46.5)	36 (36.7)	293 (46.5)	

Visual impairment	432 (60.1)	64 (65.3)	368 (59.3)	0.263

Hyperthyroidism	33 (4.5)	1 (1.0)	32 (5.1)	0.070

Parkinson disease	12 (1.6)	4 (4.1)	8 (1.3)	0.043

Diabetes	73 (10.0)	18 (18.4)	55 (8.7)	0.003

Stroke	11 (1.5)	4 (4.0)	7 (1.1)	0.026

Hip fracture in mother	93 (12.7)	12 (12.1)	81 (12.8)	0.847

Previous fracture	166 (22.7)	14 (14.1)	152 (24.0)	0.029

Previous Colles fracture	90 (12.3)	6 (6.1)	84 (13.3)	0.042

Corticosteroids	30 (4.1)	5 (5.0)	25 (4.0)	0.612

Calcium	208 (28.7)	15 (15.3)	193 (30.8)	0.002

Benzodiazepines	177 (24.3)	21 (21.2)	156 (24.8)	0.444

* Significance level corrected for multiple comparison was set at 0.0001.

The comparison of the main characteristics by gender showed significant differences in age, weight, height, smoking, coffee and alcohol consumption, but not in sun exposure, morphotype, presence of previous fractures and calcium intake.

*Table 2* presents the prevalence of osteoporosis, both overall and by gender. The prevalence of densitometric osteoporosis was three times larger in lumbar spine (35.7%), than in femoral neck 8.9%, thus driving the overall prevalence of osteoporosis: 39.4% (95% CI 35.8 – 42.9). By gender, the prevalence of osteoporosis was higher in women [43.8% (95% CI 38.8 – 49.3)] than in men [11.1% (95% CI 5.5 – 19.9)]. This difference between genders was more pronounced in the lumbar spine (men 4.1% vs women 40.5%) than in femoral neck (men 8.1% vs women 9.0%). Similarly, the presence of osteoporosis increased with age in men and women (*Table 3*). The age at which men started losing BMD was later than in women, thus causing a difference in prevalence from the younger strata onwards (men 7.8% vs. women 48.5% in the 61–75 years old stratum; and men 22.7% vs women 62.7% among 75 or above). The prevalence in women under 61 was 28.3%. There were no men under 60.

**Table 2.** Prevalence of osteoporosis, overall and by gender, in a sample of physically active Majorcan elderly.

**Bone mineral density**	**Total n=731**	**Men n=99**	**Women n=632**

Lumbar spine			
Normal	28.9 (25.1 – 33.1)	61.8 (47.2 – 79.6)	23.8 (20.2 – 28.0)
Osteopenia	35.4 (31.2 – 40.0)	34.0 (23.4 – 47.8)	35.6 (31.1 – 40.6)
Osteoporosis	35.7 (31.5 – 40.3)	4.1 (0.01 – 10.5)	40.5 (35.7 – 45.8)

Femoral neck			
Normal	42.1 (37.5 – 47.1)	42.4 (30.6 – 57.3)	42.1 (37.2 – 47.5)
Osteopenia	49.0 (44.0 – 54.3)	49.5 (36.6 – 65.4)	48.9 (43.6 – 54.6)
Osteoporosis	8.9 (6.9 – 11.3)	8.1 (3.5 – 15.9)	9.0 (6.8 – 11.7)

Any location			
No osteoporosis	60.6 (55.1 – 66.5)	88.9 (71.3 – 109.5)	56.2 (50.5 – 62.3)
Osteoporosis	39.4 (35.0 – 44.2)	11.1 (5.5 – 19.9)	43.8 (38.8 – 49.3)

**Table 3.** Prevalence of densitometric osteoporosis by age and gender strata.

	**Age groups**		
**Men**	**<60 (n=0)**	**61–75 (n=77)**	**≥75 (n=22)**

Lumbar spine			
Normal		62.7 (46.0 – 83.3)	59.1 (31.5 – 1.0)
Osteopenia		34.7 (22.6 – 50.8)	31.8 (12.8 – 65.5)
Osteoporosis		2.7 (0.3 – 9.6)	9.1 (1.1 – 32.8)

Femoral neck			
Normal		49.3 (34.9 – 67.7)	18.2 (4.9 – 46.5)
Osteopenia		45.4 (31.7 – 63.2)	63.6 (34.8 – 106.8)
Osteoporosis		5.2 (1.4 – 13.3)	18.2 (4.9 – 46.5)

Any location			
No Osteoporosis		92.2 (72.0 – 116.3)	77.3 (45.0 – 123.7)
Osteoporosis		7.8 (2.8 – 17.0)	22.7 (7.4 – 53.3)
**Women**	**≤ 60 (n=194)**	**61–75 (n=371)**	**≥75 (n=67)**

Lumbar spine			
Normal	33.2 (25.5 – 42.3)	19.5 (15.3 – 24.6)	20.9 (11.4 – 35.1)
Osteopenia	39.4 (31.0 – 49.3)	35.2 (29.4 – 41.8)	26.9 (15.9 – 42.4)
Osteoporosis	27.5 (20.6 – 35.9)	45.2 (38.6 – 52.7)	52.2 (36.4 – 72.6)

Femoral neck			
Normal	60.8 (50.3 – 72.8)	36.9 (31.0 – 43.6)	16.4 (8.2 – 29.4)
Osteopenia	36.1 (28.1 – 45.6)	53.9 (46.7 – 61.9)	58.2 (41.4 – 79.6)
Osteoporosis	3.1 (0.01 – 0.07)	9.2 (6.3 – 12.8)	25.4 (14.8 – 40.6)
Any location			
No Osteoporosis	71.6 (60.2 – 84.6)	51.5 (44.4 – 59.3)	37.3 (24.1 – 55.1)
Osteoporosis	28.3 (21.3 – 36.9)	48.5 (41.7 – 56.1)	62.7 (45.2 – 84.7)

### Determinants in men

In the bivariate model, the presence of osteoporosis in men was associated with lower weight (OR=0.89, p=0.008), lower height (OR=0.82, p=0.002), and a diagnosis of Parkinson’s disease (OR=9.55, p=0.033). In addition, an athletic morphotype was shown as a protective factor for osteoporosis (OR=0.15, p=0.023). According to the results of the multivariate analysis, the only determinants of osteoporosis were lower weight (OR=0.85; p=0.004), and an athletic morphotype (OR=0.05; p=0.004). These effects were independent of age (*Table 4*).

**Table 4.** Risk factors for osteoporosis in men, bivariate and multivariate analysis.

**Variable**	**Bivariate OR [CI95%] (p value)**	**Multivariate OR [CI 95%] (p value)**

Age	1.10 [0.99–1.23] (0.087)	1.10 [0.96–1.28] (0.174)

Weight	0.89 [0.81–0.97] (0.008)	0.85 [0.77–0.95] (0.004)

Size	0.82 [0.72–0.93] (0.002)	

Smoking habit	0.49 [0.06–4.09] (0.507)	

Morphotype		
Pyknic	1	
Asthenic	2.07 [0.31–13.7] (0.450)	0.33 [0.03–3.83] (0.375)
Athletic	0.15 [0.03–0.77] (0.023)	0.05 [0.006–0.37] (0.004)

Alcoholic habit	0.53 [0.11–2.62] (0.436)	

Coffee intake	0.62 [0.17–2.20] (0.460)	

Sun exposure	0.78 [0.15–4.03] (0.767)	

Physical Activity		
No	1	
Mild	0.92 [0.09–8.81] (0.941)	
High	1. 5 [0.15–14.55] (0.727)	

Standing upright >4 h	0.86 [0.23–3.18] (0.821)	

Visual impairment	1.48 [0.36–5.97] (0.585)	

Previous Colles fracture	-	

Hip fracture in mother	1.73 [0.33–9.18] (0.518)	

Previous fracture	1.41 [0.27–7.31] (0.685)	

Hyperthyroidism	-	

Parkinson disease	9.55 [1.20–76.2] (0.033)	

Diabetes	1.01 [0.20–5.15] (0.987)	

Stroke	2.83 [0.27–29.9] (0.386)	

Corticosteroids	-	

Benzodiazepines	0.81 [0.16–4.05] (0.795)	

Calcium	1.26 [0.24–6.53] (0.779)	

### Determinants in women

In the bivariate model, the presence of osteoporosis in women was related to age (OR=1.05; p= <0.001), lower weight (OR=0.96; p<0.001), shorter height (OR=0.93; p<0.001), and breastfeeding (OR=1.38; p=0.049). Prolonged menstruation (or late menopause) was a protective factor of osteoporosis (OR=0.96; p=0.010). Alcohol intake was found associated with osteoporosis as well (OR= 0.44, p= 0.008) (*Table 5*).

**Table 5.** Risk factors for osteoporosis in women.

**Variable**	**Bivariate OR [CI 95%] (p value)**	**Multivariate OR [CI 95%] (p value)**

Age	1.05 [1.04–1.08] (<0.0001)	1.06 [1.04–1.08] (<0.0001)

Weight	0.96 [0.94–0.98] (<0.0001)	0.97 [0.94–0.99] (0.002)

Size	0.93 [0.90–0.95] (<0.0001)	0.96 [0.92–0.98] (0.002)

Smoking habit	0.53 [0.29–0.97] (0.039)	

Morphotype		
Pyknic	1	
Asthenic	1.47 [0.84–2.53] (0.173)	
Athletic	0.73 [0.53–1.03] (0.076)	

Alcohol habit	0.44 [0.24–0.80] (0.008)	0.36 [0.18–0.72] (0.003)

Coffee intake	0.80 [0.58–1.09] (0.160)	

Sun exposure	1.37 [0.97–1.94] (0.073)	1.43 [0.98–2.09] (0.066)

Physical activity		
No	1	
Mild	1.69 [0.94–3.05] (0.080)	
High	1.65 [0.94–3.02] (0.102)	

Standing upright >4 h	1.13 [0.81–1.59] (0.463)	

Visual impairment	1.32 [0.95–1.82] (0.094)	

Previous Colles fracture	1.33 [0.84–2.11] (0.222)	

Hip fracture in mother	1.08 [0.68–1.74] (0.719)	

Previous fracture	1.39 [0.96–2.00] (0.079)	

Hyperthyroidism	1.94 [0.94–4.00] (0.073)	2.78 [1.25–6.17] (0.012)

Parkinson disease	0.42 [0.08–2.11] (0.294)	

Diabetes	1.29 [0.73–2.28] (0.378)	

Stroke	3.23 [0.62–16.80] (0.162)	

Corticosteroids	1.98 [0.88–4.48] (0.100)	

Benzodiazepines	1.27 [0.88–1.82] (0.199)	

Calcium	1.03 [0.73–1.45] (0.856)	

Pregnancy	0.75 [0.47–1.18] (0.216)	

Breastfeeding	1.38 [1.00–1.91] (0.049)	

Early menopause	1.23 [0.86–1.75] (0.247)	

Menstruating years	0.96 [0.93–0.99] (0.010)	0.96 [0.93–0.99] (0.008)

Oophorectomy	1.01 [0.61–1.68] (0.950)	

In the multivariate analysis, the main determinants of osteoporosis were increasing age (OR=1.06, p<0.0001) and the presence of hyperthyroidism (OR=2.78, p=0.012). In addition, factors associated with lower likelihood of osteoporosis were: greater weight (OR=0.97; p=0.002), higher height (OR=0.96; p=0.002), any alcohol consumption (OR=0.36; p=0.003) and later menopause (OR=0.96; p=0.008). The observed effects were independent of sun exposure.

## DISCUSSION

We have studied osteoporosis from an epidemiological perspective in a sample of physically active senior citizens from a Mediterranean area and found a larger than expected rate of densitometric osteoporosis, especially in women.

In 1995, Melton et al. estimated the prevalence of osteoporosis according to WHO criteria in 15% for white women over 50 years of age when measured in one of the three usual locations (spine, hip or wrist) and 30% when measured in all of them.^11^ In a previous study, Díaz Curiel et al. estimated the prevalence of osteoporosis in women over 50 in Spain in 26% (95% CI, 23 – 30%).^12^ In our sample, the prevalence of osteoporosis in women was 44%, larger than the estimates from Melton in 1995 and Díaz Curiel for Spain. This finding is in disagreement with estimates of osteoporotic hip fracture incidence in Spain, which show a moderate to low risk of fracture in Balearic Islands, both in men and in women.^13,14^ A plausible explanation is that other risk factors, besides BMD, are playing a protective role, probably physical activity, and mild weather, which elude falls.^15^

Male osteoporosis represents a major and growing health problem that goes under-diagnosed in the general population. It is characterised by higher morbidity and mortality compared to female osteoporosis and a high prevalence of secondary osteoporosis, between 40 and 60%.^16,17^ The most important causes of male osteoporosis are those associated with excessive alcohol intake, glucocorticoid use, and primary or secondary hypogonadism.^2^ In our study, the prevalence of osteoporosis in men was 11%, a figure that is similar to others found in the literature.^3^ Interestingly, an athletic phenotype was associated with less prevalence, a way to measure the effect of prolonged healthy lifestyles in a single variable.

Aging is, together with gender, the strongest independent determinant of osteoporosis.^18,19^ The association of age with decreased BMD has been attributed to low protein intake,^20^ low 25 (OH)D3,^21^ hypogonadism,^22^ and a reduced bone turnover rates.^16^ Also, having a previous fracture is a risk factor more common in the elderly than in the young,^23^ because age contributes to fracture risk independently of BMD, probably in relation to a higher risk of falls. To illustrate this, a Swedish study showed that, given a fixed T-score of −2.5 SD, the 10-year probability of hip fracture varies up to 5-fold depending on age.^24^

In women, menopause adds a sharp effect to that of age on BMD. Some studies show that the prevalence of osteoporosis in women increases from 15% in the 50–59 decade to over 80% in women above 80.^2,25^ Similar to previous reports, our study showed an increasing prevalence of osteoporosis increases with age, and differences by gender,^26^ with a 1:4 men/women ratio. None of the variables measured in both groups differed substantially to attribute differences to these; on the contrary, if any, more men had osteoporosis risk factors than women. This means that either gender or other unmeasured variables are directly accounting for these differences.

Measuring the prevalence of densitometric osteoporosis have led to conflicting results. The main reason is the double debate about whether BMD must be measured only at the femoral neck, or also at the spine and the total hip, and about whether a male or female reference must be used.^1^ The WHO supports the measurement of BMD at the femoral neck and the use of the cut-off value established for women (T-score of −2.5 calculated with the young white female normal reference database).^1^ Osteoporosis is a multifactorial disorder associated with low bone mass and enhanced skeletal fragility. One of this risk factors is low BMI, but, paradoxically, obesity has also seen associated to fracture.^27^ In our population, this could explain, on the one hand, the high percentage of a fracture history in any location (23%), and on the other hand, the protective effect of the athletic morphotype found in males (OR= 0.07).

We detected hyperthyroidism as strong risk factor for osteoporosis (OR= 2.8). Hyperthyroidism is known to be associated with loss of BMD and increased fracture risk, with a decline of fracture risk after adequate treatment.^28,29^ In a review, it was advocated that patients with (subclinical) hyperthyroidism should have BMD measured and, in case of reduced BMD, be offered antithyroid drugs.^30^ Hyperthyroidism should thus be taken into account when evaluating a patient with osteoporosis, not only for indicating antiosteoporotic treatment, but also for other preventive treatments.

The relationship between alcohol consumption and the presence of osteoporosis is puzzling. Most literature has found an association with alcohol consumption, and in fact, alcohol is a well-accepted risk factor for fracture and osteoporosis.^3,4,9,25,31^ On the other hand, our study shows a negative association with osteoporosis in women, and moderate alcohol intake has been positively associated with BMD and less bone loss in other studies, alluding to secretion of calcitonin or endogenous oestrogens.^31^ We cannot discard other unmeasured aspects related to a moderate intake of alcohol in women, but perhaps it has to do with an otherwise healthy lifestyle, as wine has been traditionally included as part of a Mediterranean standard of living.^9^

## LIMITATIONS

Our study has some limitations; first of all, the definition of osteoporosis in the sole basis of BMD. A percentage of individuals will suffer a fragility fracture, particularly hip or spine, without having densitometric osteoporosis, ie, being above a T-score cut-off, and should be considered to have osteoporosis and warrant treatment.^32^ more concretely, many women will suffer fractures despite only osteopenic bone mass range. Unfortunately, fractures were not included in the definition of osteoporosis in the present study for logistical reasons. As a result, our estimation of osteoporosis may be an underestimate of the true clinical burden of osteoporosis in elderly Majorcan population. Likewise, we may be underestimating the number of people who warrant treatment, as clinical decisions regarding treatment include consideration of other risk factors. A second limitation is not having men below the age of 60 and yet having postmenopausal women. This may have to do with cultural reasons and differences in healthy behaviours between genders, and very likely to not having as much spare time to get a check-up before retirement age in men. By presenting results separated by sex, we hope to override this limitation.

The uniqueness of our study is having used as target population physically active senior citizens: a common and challenging clinical scenario nowadays. Despite being an “apparently healthy and fit” sample, it presents a high prevalence of osteoporosis, which reinforces the idea that osteoporosis is a silent disease, whose consequences (fracture) shove sufferers to a status of significant physical limitation and associated morbidity, if not prevented. Since the study ended, we have been following the rate of hip fracture in the area and will try to understand whether the prevalence of densitometric osteoporosis, as high as it is in our population, is as strong a determinant in a physically active aging population as in others found in the literature. Meanwhile, we can not strongly recommend detection methods at the population level despite the high prevalence found. Also, no generalisation to the people of Majorca should we made, due to the selection scheme used (fitness setting).

## CONCLUSION

In conclusion, the prevalence of osteoporosis, as defined by densitometric cut-offs, in a physically active population of Majorcan elderly is higher than expected, especially in women; further prospective studies are needed to disentangle the outcome of a low BMD in such a population.
